# Metabolite profiling and nephroprotective potential of *Glycyrrhiza glabra* L. roots against cisplatin-induced nephrotoxicity *in vitro* and *in vivo*

**DOI:** 10.22038/IJBMS.2022.65478.14404

**Published:** 2022-11

**Authors:** Parakh Basist, Sultan Zahiruddin, Mohammad Umar Khan, Gaurav Gautam, Bisma Jan, Mohammad Ahmed Khan, Rabea Parveen, Sayeed Ahmad

**Affiliations:** 1 Centre of Excellence in Unani Medicine (Pharmacognosy and Pharmacology), Jamia Hamdard, New Delhi, India-110062; 2 Department of Pharmacology, School of Pharmaceutical Education and Research, Jamia Hamdard, New Delhi, India-110062; 3 Bioactive Natural Product Laboratory, Department of Pharmacognosy and Phytochemistry, School of Pharmaceutical Education and Research, Jamia Hamdard, New Delhi, India-110062; 4 Department of Food Technology, School of Interdisciplinary Science and Technology, Jamia Hamdard, New Delhi, India-110062; 5 Department of Pharmaceutics, School of Pharmaceutical Education and Research, Jamia Hamdard, New Delhi, India-110062

**Keywords:** Cisplatin, Glycyrrhizin, Glycyrrihza glabra, Kidney disorder, Nephroprotective, Nephrotoxicity

## Abstract

**Objective(s):**

The present study was conducted to investigate the phytochemical analysis and demonstrate the nephroprotective potential of root extract of *Glycyrrhiza glabra* L. against cisplatin (CP) -induced nephrotoxicity* in vitro* and *in vivo.*

**Materials and Methods:**

The HPTLC analysis and UPLC-MS were carried out for standardizing and metabolite profiling of methanolic extract of roots of *G. glabra* (GGE). Further, *in vitro *studies were conducted in human embryonic kidney (HEK)-293 cells to evaluate the cytotoxicity and anti-oxidant potential of GGE with CP as a toxicant and ascorbic acid as standard. Also,* in vivo* nephroprotective potential at doses of 31.5, 63, and 126 mg/kg/day on CP (6 mg/kg, bw, IP) induced nephrotoxicity was evaluated on rodents.

**Results:**

Phytochemical analysis by HPTLC and UPLC-MS revealed the presence of glycyrrhizin, glabridin, and liquiritin along with other bioactive constituents. The in vitro assay of GGE showed significant (*P*<0.001 nephroprotective, cellular anti-oxidant potential and improvement in morphological changes induced by CP. Further, administration of CP caused significant (*P*<0.001) elevation in biochemical, inflammatory, oxidative stress, caspase-3, as well as histopathological changes in kidney tissue. Pre-treatment with GGE attenuated the elevated biochemical markers significantly, improved histopathological damage, and showed a comparable result to ascorbic acid and α-ketoanalogue.

**Conclusion:**

Present study concluded the nephroprotective potential of GGE which supports the traditional claim of *G. glabra* roots in various kidney and its related disorders. The nephroprotective activity may be attributed to its anti-oxidant, anti-inflammatory, and anti-apoptosis effects. Thus, it holds promising potential in management of nephrotoxicity.

## Introduction

Licorice is a leguminous plant widely used as a medicinal plant in the food industry and extensively researched in herbal medicine. Licorice is known by several names including licorice, sweetwood, licorice radix, lakritzeholz (German), Meyan or Beyan (Turkish), Gan Cao (Chinese), Solodka (Russian), and reglisse (French). The name licorice essentially originated from the Greek word glykyrrhiza in which “glykys” referred to sweet whereas “rhiza” means root (1). 

Amongst thirty known varieties of licorice, *Glycyrrhiza glabra* L. is one of the most widely utilized and distributed species of licorice and is found in Italy, Spain, Turkey, Central Asia, and various parts of China. *G. glabra* is widely utilized in folk medicine as an antibacterial, antifungal, anti anti-inflammatory, anti-oxidant, anti-parasitic, antipyretic, anti spasmodic, anti -viral agent, and also as a medicament in infection and diarrhea (2). Globally, *G. glabra*’s consumption as a spice is very high due to its high flavors and textures.* G. glabra* as well as its secondary metabolites have been reported to exhibit great activity against various complications (3). Commonly, roots of *G. glabra* are employed for the preparation of tea as it has high thirst-quenching efficiency (4). Also, the dried roots have been used as a tooth cleaner. In particular, *G. glabra* is widely used in the food industry as a food additive including sweetening and flavoring agent. The root is utilized as a flavoring agent for various food-related products such as chewing gum, candies, ice cream, baked goods, soft drinks, and American-type tobacco. The root fibers are utilized in the preparation of boxboard, wallboard, and insulation material whereas root extracts are used in beer and fire extinguishers as foaming agents. In topical cosmetic products, *G. glabra *is incorporated as a skin pigmentation agent (5). 

In the traditional system, licorice roots have been utilized as medicine for more than 4000 years in various ailments including lung diseases, kidney disorders, heart diseases, arthritis, eczema, gastric ulcer, allergies, low blood pressure, liver toxicity, and microbial infections. Also, medicinal uses are recorded in ancient data and originated in Iraq. Medicinal uses of licorice are reported in pharmacopoeias from India (Ayurvedic Pharmacopoeia, 2001 and Unani Pharmacopoeia, 2007), Britain, Germany, and France are in agreement on the medicinal uses of licorice (6). The extract of this plant contains multiple anti-oxidant and anti-inflammatory phytochemicals. Key phytochemicals in *G. glabra* include saponins including glycyrrhizin which is considered a primary bioactive constituent, flavonoids, phytosterols, and minor phenolic compounds which exhibit wide biological activities (7).

The kidney is considered a major organ that carries essential functions of the body like waste product clearance, management fluid volume, maintenance in the balance of electrolytes as well as acid-base, and endocrine functions. It derives 25% of total body heat output and is also exposed to drugs and chemicals in circulation as main excretion body. The nephrotoxic drugs contribute to acute as well as chronic kidney failure and a rise in morbidity and mortality (8). Nephrotoxicity is a well-known complication induced by various anticancer drugs as well. According to current evidence, nephrotoxicity is considered the most continuing renal problem that causes lifetime risk in the world population comprising Europe 8–15%, Asia 2–5%, and the Middle East 20% (9). Cisplatin (CP) is a core drug prescribed as an anti-neoplastic drug mainly against bladder, head, lung, neck, ovarian, and testicular tumors. High doses of CP are even more efficacious, but the use of high doses can lead to severe toxicity, particularly in renal tissue. The renal toxic effects shown by CP are cumulative and dose-dependent which require either dose reduction or even withdrawal. CP leads to oxidative stress, apoptosis, as well as DNA damage. Among patients treated with CP, 20–35% develop renal disorders. Currently, no effective drug is available to prevent or treat CP-induced renal toxicity. Therefore, large numbers of natural as well as chemical compounds with anti-oxidant and anti-inflammatory properties have been evaluated for their potential effects against CP-induced nephrotoxicity. Many natural products have shown efficacy and low toxicity to protect against CP-induced renal toxicity by mainly acting as anti-oxidant and anti-inflammatory and also through restoration of anti-oxidant enzymes thus protecting against oxidative stress (10). Hence there is a need for alternative medicine which can be used as adjuvant or alone for the treatment of kidney disorders. 

Rhizomes, roots, and leaves of *G. glabra *are the most important medicinal parts which have been reported for their pleiotropic effects to be used either alone or in combination with other bioactive compounds or modern medicines. In 2018, leaf extract of *G. glabra *showed nephroprotective properties by modulating renal biochemical, structural, and hematological changes in carbon tetrachloride-induced nephrotoxicity in mice **(**11**)**. Another study reported that rhizome of *G. glabra* at doses of 100, 200, and 400 mg/kg alleviated the methotrexate-induced hepatic and renal toxicity by modulating serum, oxidative stress biomarkers, interleukins, as well as histopathological and immunohistochemical improvements **(**12**)**. Another, recent study conducted in 2020 by Man and colleagues reported hepato-protective efficacy of wild roots of *Glycyrrhiza uralensis *through anti-oxidative stress, anti-inflammation, anti-apoptosis, as well as acceleration of metabolism **(**13**)**. Recently in 2021, the study concluded the ameliorative effects of *G. glabra *root extract in gentamicin-induced nephrotoxicity in mice by increasing Gpx and SOD levels and decreasing IL-1β and IL-6 levels. Also, down-regulation of Cox-2 and Bax, up regulation of HO-1 and Nrf-2 expression, and normalization of histopathological characteristics by root extract were reported **(**14**)**. In 2017, a study was conducted in Iraq on the nephroprotective potential of leaf extract of *G. glabra* in gentamicin induced nephrotoxicity by modulating serum enzymes (AST, ALP, and ALT), renal damage markers, serum electrolytes as well as oxidative stress markers **(**15**)**. 

Considering, a number of therapeutic properties of *G. glabra *L., this plant exhibits high pharmacological and nutraceutical potential. Limited reports on the metabolite profiling of root extract of *G. glabra* are available however, nephroprotective potential of root extract was conducted against gentamicin and acetaminophen as toxicant with no data on the possible mechanism and without metabolite profiling. Hence, it was thought worthwhile to work on *G. glabra *root extract for its nephroprotective potential in rodent model against CP-induced nephrotoxicity followed by metabolite profiling to work out its mechanism and to develop a standardized extract which can be used further for drug development in AYUSH model.

Keeping this in mind, our study demonstrated complete chemical and pharmacological evaluation of the *G. glabra* root extract including its quality control, metabolite profiling using HPTLC and UPLC-MS as well as evaluation of nephroprotective effects in cell line and rodent model. Furthermore, this is the first attempt that demonstrates the nephroprotective potential of root extract of *G. glabra *L. in HEK-293 cells and rodents against CP-induced nephrotoxicity by evaluating kidney, electrolytes, liver, anti-oxidant, inflammatory, and apoptotic biochemical markers and histopathological evaluation which is worthwhile to explore the molecular mechanism of *G. glabra *L. roots in kidney disorders. 

## Materials and Methods

***Plant extraction***

The plant materials were procured from the local market of Delhi, India, and authentication was carried out as per Ayurvedic Pharmacopoeia (Anonymous, 2001). The authenticated plant material has been deposited in the Bioactive Natural Product Laboratory, Jamia Hamdard with specimen number BNPLJH/PhD/10/19/01 for future reference. For extraction, the plant material was crushed into coarse powder and macerated overnight in methanol in a 1:10 (w/v) ratio. Further, it was sonicated for 30 min at 50 **°**C and evaporated to dryness under reduced pressure. The percentage yields of extract were calculated and stored for further analysis at 4 **°C.**

***Estimation of total phenolic and flavonoid content***

The phenolic and flavonoid content of the extract was measured using Folin-Ciocalteu and aluminum chloride methods with slight modifications (16). For phenolic content, the stock solution of extract was prepared at a concentration of 10 mg/ml, then 1.0 ml of sample was added to 5.0 ml of Folin-Ciocalteu (10%) reagent followed by 4 ml of sodium carbonate (7.5%). The mixture was incubated for 40 min at room temperature for color development. Absorbance was then recorded at 765 nm. The calibration curve was prepared using Gallic acid as standard and total phenolic contents were expressed as GAE (mg/g) **(**17).

For estimation of total flavonoid content, 0.5 ml of extract sample was mixed with 0.5 ml of aluminum chloride solution in methanol (10%). The mixture was incubated for 1 hr at room temperature and absorbance was recorded at 420 nm. The calibration curve was prepared using rutin as standard and total flavonoid contents were expressed as rutin (mg/g)**(**18).

***Estimation of in vitro anti-oxidant activity***

*1-diphenyl-2-picrylhydrazyl (DPPH) assay*

Free radical scavenging potential was estimated as the earlier described method with slight modifications and ascorbic acid was adopted as the positive control **(**19**, **20). The IC_50 _(half-maximal inhibitory concentration) was calculated to express radical scavenging activity. The potential of the sample to scavenge radicals was calculated based on the following formula:

Percent inhibition of DPPH= [(A_control_ – A_sample_)/A_control_] × 100

*Reducing power assay*

The Fe^3+ ^Fe^2+^ transformation in the extract was estimated by following the standard method (21). The reducing ability serves as a significant indicator of anti-oxidant potential. The reducing capacity of extract increases with increasing concentration of extract. 

*Total anti-oxidant capacity (TAC)*

The TAC of the extract was estimated by the previously described method with slight modifications (22)1-diphenyl-2-picrylhydrazyl radical (DPPH. Ascorbic acid was utilized as a positive control and results were expressed in percentage based on the following formula:

Total anti-oxidant capacity (%) = [(A_control_ – A_sample_)/A_control_] × 100

***Quantitative estimation of glycyrrhizin in GGE***

Glycyrrhizin was used as a specific marker compound for *G. glabra, *and glycyrrhizin was quantified in GGE using HPTLC. 30 mg/ml solution of the GGE and 1 mg/ml solution of glycyrrhizin (Sigma-Aldrich, USA) were prepared in methanol. A 6 μl of GGE and 50–4000 ng/ml glycyrrhizin were applied simultaneously with a 6 mm wide band length to pre-washed and activated silica gel 60 F254 pre-coated HPTLC plates (20x10 cm; Merck, Germany) under automated pressure of nitrogen gas flow providing a delivery speed of 150 nl/s. The TLC plate was developed in a pre-saturated TLC development chamber containing toluene, ethyl acetate, and glacial acetic acid (6:3.5:0.5; v/v/v) as a solvent system. The plate was developed to a distance of 80 mm at room temperature (25 °C). The developed plate was air dried and derivatized with an anisaldehyde sulfuric acid reagent. The quantification of glycyrrhizin was carried out at 540 nm using Camag TLC scanner III using Wincats1.2.3 software (23).

***Metabolite profiling of GGE by UPLC-MS***

50 mg of GGE was dissolved in 5 ml of volumetric flasks using LC-MS grade methanol to get 10 mg/ml solutions, filtered through a 0.2-µm membrane filter. The filtered solution was diluted in a ratio of 1:10 (v/v) using methanol. High-throughput profiling of metabolite in the extract was carried out by UPLC-MS. The UPLC was performed on a Water’s ACQUITY UPLC(TM) system (Serial No # F09 UPB 920M; Model code # UPB, Waters Corp., MA, USA) equipped with a binary solvent delivery system, an auto-sampler, column manager, and a tunable MS detector (Serial No# JAA 272; Synapt; Waters, Manchester, UK) installed and controlled by Mass Lynx V 4.1 (Waters, USA). Data acquisition was done in positive modes. Chromatography was performed using acetonitrile (A) and water (B) as the mobile phase on a monolithic capillary silica-based C18 column (ACQUITY UPLC(R) BEH C18, 1.7 µm, 2.1 x 100 mm), with the pre-column split ratio 1:5 at ambient temperature. Chromatographic separation was achieved by gradient elution mode (initially, 10% A; 0–5 min 40% A; 5–10 min 60% A; 10–13 min, 90% A; 13–15 min, 100% A; 15–16 min 10% A), and the total run time was 16 min. The capillary and cone voltages were set to 3.0 and 40 KV, respectively. For collision, argon was employed at a pressure of 5.3 х 10^-5^ Torr. The flow rate of the nebulizer gas was set at 10 μl/min, for the cone gas, set to 50 L/h, and the source temperature was set at 100 °C. The cone and capillary voltages were set to 40.0 and 3.0 kV, respectively. For collision, argon was employed at a pressure of 5.3 × 10^–5^ Torr. The accurate mass and composition for the precursor ions were calculated using the Mass Lynx V 4.1 software incorporated in the instrument (23).

***In vitro cell culture studies***

*Cell line*

Human embryonic kidney-293 (HEK-293) cells were cultured in Dulbecco’s Modified Eagle Medium (DMEM) supplemented with 10% fetal bovine serum as well as antibiotics and allowed to grow in a CO_2 _(5%) incubator at a temperature of 37 °C. The cells with a confluence of 70–80% were then subcultured according to the need of the experiment. 

*Cytotoxicity study*

HEK-293 cells from a tissue culture flask were collected and utilized to prepare stock suspension and seeded in a 96-well plate along with DMEM 0.1 ml. The cell-cultured plate in absence of a sample and standard was incubated for 24 hr. Further, 100 µl of different concentrations test sample and standards (1–1000 µg/ml) and CP (0.2–200 µg/ml) were then added and incubated under CO_2_ (5%) atmosphere for 24 hr. Thereafter, 10 µl MTT reagents (5 mg/ml) were added and incubated for 4 hr. The formazan dark-blue crystals formed were allowed to dissolve in DMSO (solubilizing agent) and the absorbance was measured at 570 nm by using a microtitre plate ELISA reader (16). All the estimation measurements were observed in triplicate and the cell viability was calculated using the following formula: 

% Cell viability = × 100

*Assessment of cytoprotective assay*

The Cytoprotective assay was conducted as per earlier mentioned protocol with slight modifications. The stock solution of the sample (1 mg/ml) was prepared using DMEM and 100 µl of sample in different concentrations. 18.75–300 µg/ml were added in a well-cultured cell plate and incubated for 24 hr. Furthermore, 100 µl of CP (13 µg/ml) were added to cells, and the plate was again incubated for 24 hr. The media was replaced with 100 µl DMEM without phenol red followed by the 10 µl MTT reagent (5 mg/ml) and the plate was allowed to be incubated for 3 hr. The supernatant was removed and formazan crystals were allowed to dissolve in 100 µl of DMSO. ELISA was executed at 540 nm and the protective effect of the extract was quantified. Ascorbic acid was employed as standard control (24). 

% Cell viability = × 100

*Intracellular ROS level measurement*

To detect the cellular ROS production of extract the earlier described protocol was adopted with slight modifications (25). The pre-cultured HEK-293 cells in 96-well plates were treated with the extract (187.5–300 μg/ml) and incubated for 3 hr. After incubation cells were rinsed with 50 μl PBS (pH 8), followed by addition of peroxide-sensitive fluorescent probe 100 µl 2’,7’-dichlorodihydro fluorescein diacetate acetyl ester (H_2_DCFDA) (10 µM) in each well. The ROS production was recorded using a fluorescence microscope at 485 nm (excitation wavelength) and 530 nm (emission wavelength) (26).

*Morphological evaluation*

The HEK-293 cells were monitored microscopically using EVOS XL Core Imaging System to evaluate the morphological changes in the toxic group as well as the treatment group, including GGE and standard group (27). 

***In vivo Evaluation on CP-induced nephrotoxicity***

*Experimental Animals*

Albino Wistar female rats of 5–7 weeks weighing 200–250 g were used for *in vivo* studies. Animals were obtained from the Central Animal House Facility of Jamia Hamdard which was approved (approval number: 1829) by Institutional Animal Ethics Committee (IAEC), Jamia Hamdard, New Delhi, India (registration no.: 173/GO/RE/S/2000/CPCSEA). All the animals were housed in polypropylene cages and at standard laboratory conditions (12:12 hr light and dark cycle, 23 ± 2 °C temperature, and 55 ± 5% relative humidity). Also, throughout the experimentation, the animals were fed with normal pellet diet and had free access to water (*ad libitum*). The studies were done by following the guidelines of the CPCSEA, New Delhi, India, as well as IAEC, Jamia Hamdard, New Delhi, India. 

*Experimentation design*

**Figure 1:**
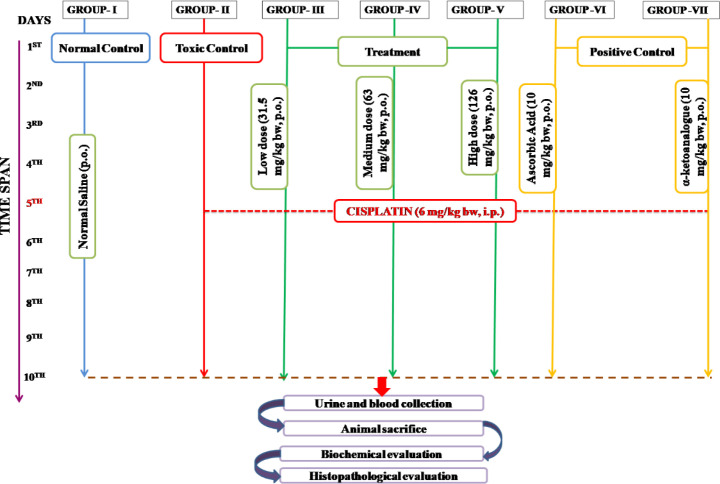
Experimental design and protocol of Wistar rats divided into 7 groups (n=8) for 10 days to demonstrate the nephroprotective potential of GGE in CP--d nephrotoxicity

GGE: *Glycyrrhiza glabra *extract*; *CP: Cisplatin

56 male Wistar rats were randomly divided into seven equal groups (Figure 1). The dose and schedule of CP administration adopted in this study were based on the method reported in the literature (28). Three dose levels, i.e., low, medium, and high calculated from the Ayurvedic pharmacopoeia of India were used for the present study. Rats in group I served as normal control (NC) administered with normal saline orally for 10 successive days while group II served as toxic control (TC) injected with a single dose of CP alone (6 mg/kg bw, IP) on the 5th day only. The 3^rd^, 4^th^, and 5^th^ groups, i.e, Group III, IV, and V served as treatment groups administered with low (31.5 mg/kg/day bw, PO) (LD), medium (63 mg/kg/day bw, PO) (MD), and high (126 mg/kg/day BW, PO) (HD) doses of GGE for 10 days from 1^st^ to 10^th^ day. Rats in Groups VI and VII served as positive control groups administered with ascorbic acid (PC-I) and α-ketoanalogue (PC-II) both at a dose of 10 mg/kg/day bw, PO for 10 successive days. Also, treatment (G-III, IV, and V) and standard (G-VI and VII) groups were injected with a single dose of CP (6 mg/kg bw. IP) on the 5th day of study. The body weight of all rats was measured at day 0 followed by once every week as well at the end of the experiment. After 24 hr completion of dosing, animals were anesthetized for blood collection followed by separation of serum for biochemical estimation. Further, animals were sacrificed to collect kidney tissue to assess oxidative parameters and histopathological examination. 

*Evaluation of kidney and hepatic biomarkers*

Different biochemical markers of the kidney, as well as the liver, were analyzed by urine and serum sample. Various kidney biomarkers including creatinine (Cr), blood urea nitrogen (BUN), uric acid (UA), total protein (TP), total bilirubin (TB), direct bilirubin (DB), albumin (Alb), globulin (Glb), calcium (Ca), sodium (Na), potassium (K), and phosphorus (P) levels were analyzed in serum and urine whereas hepatic biomarkers including alanine aminotransferase (ALT), alkaline phosphatase (ALP), and aspartate aminotransferase (AST) were analyzed in serum (29).

*Evaluation of oxidative stress and anti-oxidant biomarkers in kidney*

Kidney tissue collected from each rat and homogenates were prepared in tris-phosphate buffer (50 Mm, pH 7.4) followed by 10 min of centrifugation at 1300 rpm and temperature of 4 °C. Further, the supernatant was utilized for analysis of superoxide dismutase (SOD), nitric oxide (NO), catalase (CAT), malondialdehyde (MDA), glutathione (GSH), and glutathione peroxidase (GPx) levels by spectrophotometric assays (30).

*Evaluation of pro-inflammatory cytokines in the kidney*

TNF-α, as well as IL-1β, were measured by an enzyme-linked immunosorbent assay (ELISA) as per the protocol of the manufacturer using an ELISA reader (31).

*Evaluation of caspase-3 activity*

Caspase-3 was determined using renal tissue homogenate by following the procedure of a commercial ELISA kit according to the manufacturer (31).

*Histopathological evaluation*

The histopathological kidney analysis was performed and photographs were taken using an Olympus light microscope (IX 71) with an Olympus digital camera. Firstly, the kidney of rats from all groups was removed and allowed to fix in a freshly prepared neutral buffer. Furthermore, the paraffin sections were prepared and 5 µm thick sections were cut on a rotary microtome. The sections were then stained using the hematoxylin-eosin dye and mounted with Canada balsam (32).

## Results

The authenticated plant material was extracted through maceration using methanol for 24 hr followed by sonication. The percentage yield of the extract was 18.62 ± 1.38%.

***Phenolic and flavonoid contents of GGE***

Phenolic and flavonoids are considered important secondary metabolites having a wide range of pharmacological activities and health benefits. Folin-Ciocalteu and aluminum chloride methods have been adopted for measuring total phenolic and flavonoid contents, respectively. TPC was found to be 17.53 ± 0.08 mg of Gallic acid equivalent (GAE) per gram dry weight of extract calculated from the calibration curve with R^2^=0.996 whereas TFC was calculated from the calibration curve of rutin R^2^= 0.994, and found to be 8.24 ± 0.07 mg of rutin equivalent per gram dry weight of the extract.

***Anti-oxidant potential of GGE***

To evaluate the anti-oxidant characteristics of GGE free radical scavenging assay was conducted. IC_50_ (µg/ml) of extract and standard drug ascorbic acid was found to be 52.75 ± 0.08 and 44.52 ± 0.70, respectively. Further, a reducing power assay was also performed to investigate the ferricyanide into ferrocyanide transformations of extract. The reducing power of the extract was found to be concentration dependent with an IC_50 _value of 67.49 ± 0.52 and 52.86 ± 2.63 for standard ascorbic acid. Lastly, TAC which in presence of anti-oxidant metabolites leads to the reduction of phosphomolybdate ion and forms green phosphate complex was measured spectrophotometrically. The TAC of the extract was found to be 83 ± 1.82 mg equivalent of ascorbic acid.

***Quantitative estimation of glycyrrhizin in GGE***

Quantitative estimation of glycyrrhizin in GGE was performed using HPTLC. The results of the study showed that the developed and validated method was found linear, accurate, and precise against the different concentrations of glycyrrhizin ranging from 50–4000 ng/ml. The linear regression calibration curve was plotted between area vs concentration and found linear for glycyrrhizin (R^2^=0.999). The average LOD and LOQ for the developed method were found as 6.77 ± 0.23 and 20.52 ± 0.81 ng/spot, respectively. The intra-day and inter-day precision were determined as percentage relative standard deviation (%RSD) or the coefficient of variation and the results showed intraday and intraday precisions of 0.93–2.98 and 0.92–2.79, respectively. The accuracy of the developed method was determined based on percentage drug recovery by percentage spiking with 0, 50, 100, and 150% of the standard to the sample. The exhibited percentage recovery for glycyrrhizin was found in the range of 97.99–98.22%. The content of glycyrrhizin was found 20.95 ± 0.62 µg/mg of the sample. The HPTLC plate and their chromatogram have been summarized in supplementary Figure 1.

***Identification of metabolites in GGE by UPLC-MS***

**Figure 2:**
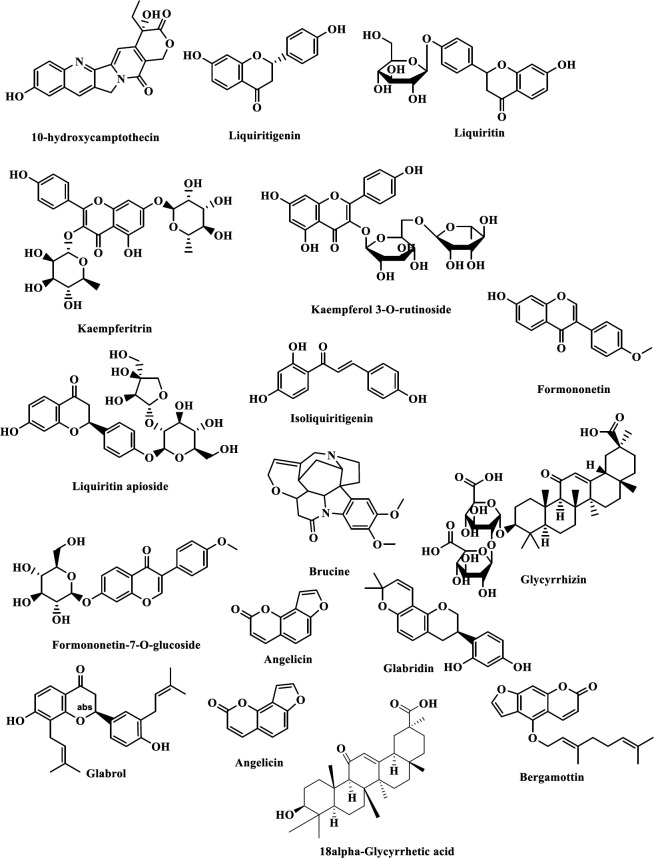
Structures of identified metabolites in *Glycyrrhiza glabra *extract using UPLC-MS

It is essential in order to characterize the bioactive compounds of extracts. The highly abundant metabolites of GGE through UPLC-MS are summarized in Table 1 and their structures in Figure 2. Most of the identified metabolites of GGE are previously reported in *G. glabra *(33)(34). The identified metabolites belong to different chemical classes. Some major metabolites of GGE are glycyrrhizin, glabridin, liquiritin, liquiritigenin, liquiritin apioside, isoliquiritigenin, glabrol, and 18 alpha-glycyrrhetic acid (35). The chromatogram of GGE has been shown in supplementary Figure 2. 

***Nephroprotective effect of GGE in HEK-293 cells***

*Cytotoxicity studies of GGE*

The *in vitro *studies were conducted in HEK-293 cells to evaluate the cytotoxicity, nephroprotection, and cellular anti-oxidant potential of GGE. In the cytotoxicity study, the IC_50_ of GGE was calculated and found to be 280.05 ± 5.21 µl/ml. Thus, the concentration 300 µl/ml was selected to evaluate the nephroprotection, cellular anti-oxidant activity, and morphological improvement in HEK-293 cells.

*Effect of GGE against CP-induced toxicity*

**Figure 3:**
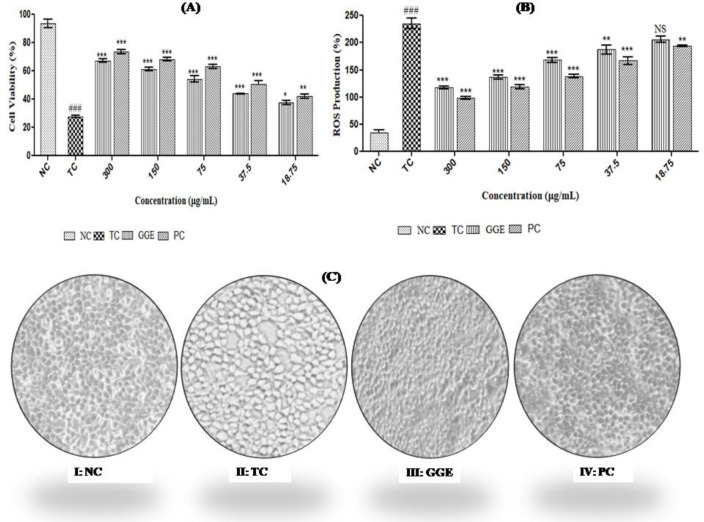
Effect of GGE in CP-induced toxicity *in vitro* using HEK-293 cells A: Nephroprotective effect of GGE and standard in CP-induced cytotoxicity, B: Anti-oxidant potential of GGE and standard in CP-induced ROS increase, and C: Morphological changes in HEK-293 cell line

The nephroprotective effect of GGE was evaluated by CP-induced toxicity in HEK-293 cells. The experimental data revealed a significant reduction (*P*<0.001) in cell viability which was reversed when co-treated with GGE as well as standard as shown in Figure 3A.

*Cellular anti-oxidant effect of GGE*

DCFH-DA fluorescent probe was used to evaluate the *in vitro* anti-oxidant potential of GGE against CP-induced oxidative stress via intracellular ROS production in HEK-293 cells. The experimental study revealed the increased levels of ROS in the toxic group, i.e., CP incubated group as compared with the control group. Although treatment with GGE at a concentration of 18.75–300 µg/ml was found to decrease CP-induced ROS production in a dose-dependent manner, no significant differences were observed in comparison to the standard as shown in Figure 3B. 

*Effect of CP, GGE, and standard on the morphology of HEK-293 cells*

Changes in the morphology of HEK-293 cell lines have been observed in different groups including control, CP-induced toxicity, as well as treatment groups both GGE and standard. The Control group showed normal architecture of cells, i.e., small, polygonal-shape as well as epithelial-like structure. Cells exposed to toxicant CP leads to distortion in the architecture of the cell with a spherical and rounded shape. Also, cytoplasmic vacuolation was observed when compared with the normal control group as shown in Figure 3 (C).

***Nephroprotective effect of GGE in CP-induced nephrotoxicity in Wistar rats***

*Body weight changes*

The effect of administration of CP showed significant (*P*<0.01) loss in weight as compared with the control group. Interestingly, administration of GGE elicited a significant increment in body weight of rats G-III (LD) (*P*<0.01), G-IV (MD) (*P*<0.001), and G-V (HD) (*P*<0.001) as compared with G-II (TC). However, treatment with standard also resulted in a significant (*P*<0.01) increase in body weight as compared with G-II (TC).

*Effect of GGE on biochemical markers*

The data reveals that CP causes significant changes in levels of liver and kidney biomarkers. In G-II (TC), CP leads to a significant (*P*<0.001) increase in the levels of Cr, BUN, urea, and uric acid in the serum of the G-II (TC) as compared with G-I (NC) group, while the change has been significantly (*P*<0.001) reversed in the treatment group (GGE). Further, serum levels of Alb and TP were significantly decreased (*P*<0.001), whereas Glb, DB, and TB and urine levels of Alb and TP were significantly increased (*P*<0.001) as compared with G-I (NC). In addition, serum electrolyte levels were decreased significantly (*P*<0.001) and urine electrolyte levels were increased significantly (*P*<0.001) as compared with G-I (NC). These alterations in levels of kidney and liver function parameters confirmed the CP-induced nephrotoxicity. However, pre-treatment of GGE at different doses resulted in significant improvement in levels of kidney and liver function biomarkers as compared with G-II (TC) as given in Table 2.

*Effect of GGE on oxidative-stress parameters*

**Figure 4:**
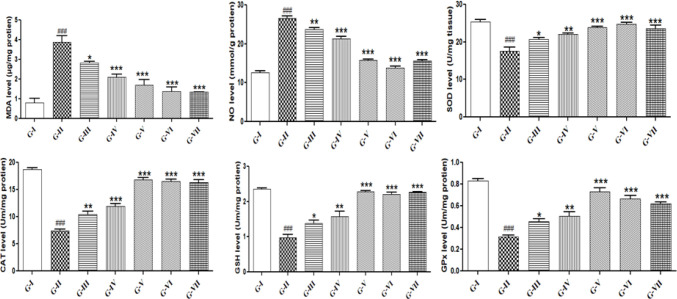
Anti-oxidant potential of GGE in CP-induced nephrotoxicity

The levels of MDA and NO in the tissues of CP-induced rats were significantly higher, while the levels of SOD, CAT, GSH, and GPx were significantly (*P*<0.001) lower as compared with G-I (NC). Pre-treatment with different doses of the extract showed significantly (*P*<0.001, *P*<0.01, and *P*<0.05) improved levels of both enzymatic as well as non-enzymatic anti-oxidants as compared with G-II (TC) (Figure 4).

*Effect of GGE on inflammatory and apoptosis (caspase-3) markers*

**Figure 5:**
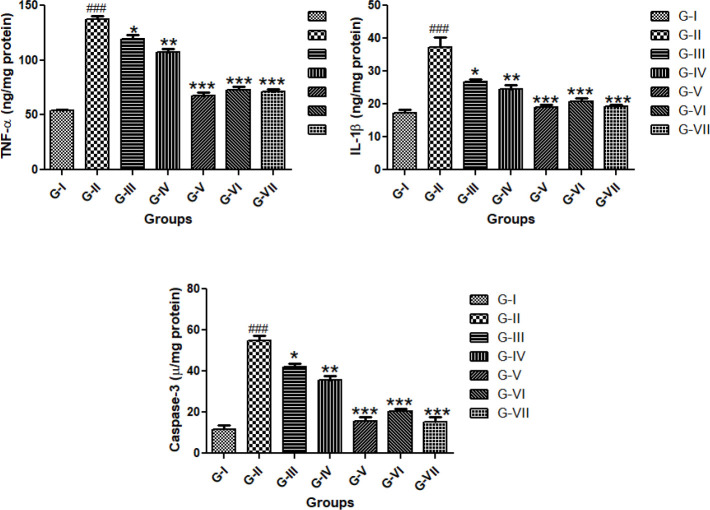
Inflammatory and apoptotic (caspase-3) potential of GGE in CP-induced nephrotoxicity

Induction of CP showed significant (*P*<0.001) higher levels of TNF-α and IL-1β as well as caspase-3 as compared with the G-I (NC). However, different doses of extract ameliorate CP-induced inflammatory stress as well as apoptosis by significant (*P*<0.001, *P*<0.01, and *P*<0.05) alteration (Figure 5).

*Effect of GGE on histopathological changes on kidney*

**Figure 6:**
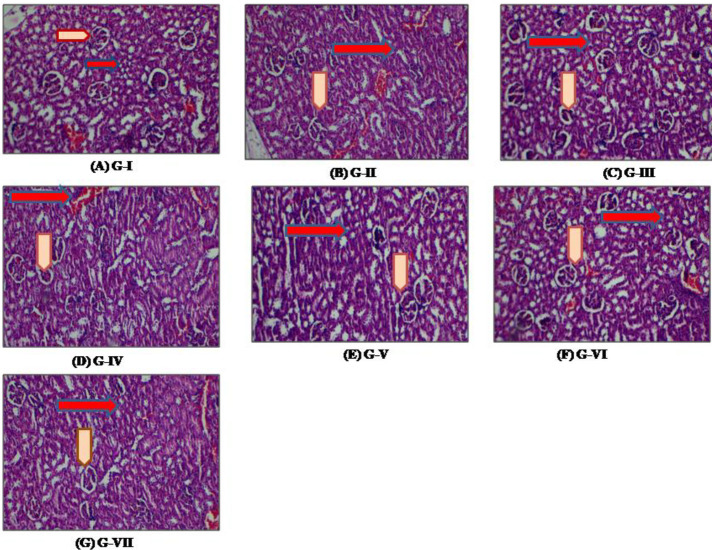
Histopathological changes in kidney tissue in different animal groups: A: photomicrographs of G-I (Normal Control) shows normal glomeruli (arrowheads) as well as tubules (arrows) along with no abnormalities; B: photomicrographs of G-II (Toxic Control) of rat treated with CP shows abnormality in the tubule infiltrate (arrows) and interstitium (arrowheads); C: photomicrographs of kidney of G-III (Treatment-Low dose) treated with CP and low-dose of extract shows minimal tubular injury (arrowheads) and interstitial capillaries congestion (arrow); D: G-IV (Treatment-Medium dose); E: G-V (Treatment-High dose): photomicrographs of kidney treated with CP and medium- and high-dose of extract shows normal kidney morphology interstitial capillaries (arrows) and mild degenerating tubules (arrowheads); F: G-V (Treatment-High dose) and G: G-VII (Positive control-II) photomicrographs of kidney treated with CP and standard shows normal cellular morphology with minimal tubular necrosis

Histological changes in Wistar rat renal tissues which occur as a response to CP and GGE are shown in Figure 6. The kidneys harvested from all groups were studied using a light microscope. The four compartments including blood vessels, glomeruli, interstitium, and tubules were studied for histopathological changes in the kidney. In G-I (NC), no histopathological changes in blood vessels, glomeruli, interstitium, and tubules were observed. The CP-induced group G-II (TC) showed histopathological abnormalities in interstitium as well as tubules infiltrate. Mildly chronic patchy lymphoplasmacytic inflammatory cell infiltrates as well as mild congestion in interstitium were observed. Besides, acute patchy tubular injury with reactive atypia of epithelial cells in tubules was observed. Also, in some tubular epithelial cells, cytoplasmic vacuolization, as well as apoptosis, were seen. Blood vessels and glomeruli were not affected in G-II (TC). G-III (LD) showed normal glomerulus and minimal tubular injury of only a few tubules as well as little congestion in interstitial capillaries. However, normal morphology of the kidney with mild degenerating tubules was seen in G-IV (MD) and G-V (HD) groups. Both G-VI and G-VII (PC-I and PC-II) revealed normal cellular morphology of the kidney with little tubular necrosis.

**Table 1:** Identified metabolites in *Glycyrrhiza glabra* extract using Ultra-high performance liquid chromatography- mass spectrometry (UPLC-MS)

**Comp. No.**	**R**_t_	**M/Z theoretical**	**M/Z practical**	**Compound**	**Mass ID**
1	0.623	364.360	365.168	10-hydroxycamptothecin	MassBank Record: NGA02746
2	3.034	256.260	257.222	Liquiritigenin	MassBank Record: NGA03254
3	3.034	578.163	579.144	Kaempferitrin	MassBank Record: NA002999
4	3.034	594.522	595.147	Kaempferol 3-O-rutinoside	MassBank Record: PM000504
5	3.152	418.398	419.231	Liquiritin	MassBank Record: PR303642
6	3.152	550.513	551.170	Liquiritin apioside	MassBank Record: PM019119
7	4.298	256.250	257.096	Isoliquiritigenin	PubChem CID: 638278
8	4.298	430.409	431.140	Formononetin-7-O-glucoside	MassBank Record: PR302340
9	6.017	394.189	394.342	Brucine	MassBank Record: NA002556
10	6.017	268.268	269.196	Formononetin	MassBank Record: PR302848
11	6.017	822.942	823.596	Glycyrrhizin	MassBank Record: PS044005
12	6.017	394.189	394.342	Brucine	MassBank Record: NA002556
13	7.585	186.168	187.080	Angelicin	MassBank Record: NGA03270
14	8.832	324.376	325.154	Glabridin	MassBank Record: PM019129
15	9.776	392.495	393.271	Glabrol	MassBank Record: PM019130
16	10.956	186.168	186.954	Angelicin	MassBank Record: NGA03270
17	10.956	338.151	339.017	Bergamottin	MassBank Record: BML00648
18	11.546	470.698	471.466	18alpha-Glycyrrhetic acid	MassBank Record: NGA02587
19	12.490	424.541	425.343	Polyanthinin	MassBank Record: NGA00031

One-way ANOVA was used for statistical analysis in A and B. TC was compared with NC, ###*P*<0.001, ##*P*<0.01, #*P*<0.05, and GGE and PC were compared with TC. ***P<0.001, **P<0.01, and *P<0.05 were considered significant and NS: non-significant

NC: Normal control; TC: Toxic control; GGE: *Glycyrrhiza glabra *extract; PC: Positive control

**Table 2:** Biochemical parameters in serum and urine for the nephroprotective activity of *Glycyrrhiza glabra* extract against CP-induce nephrotoxicity

	**Animal grouping**							
**Parameters**		**G-I**	**G-II**	**G-III**	**G-IV**	**G-V**	**G-VI**	**G-VII**
**Kidney function test**								
**Creatinine (mg/dl)**	**Serum**	0.87±0.05	3.305±0.15^###^	2.608±0.13^NS^	1.383±0.16^***^	2.230±0.06^**^	1.473±0.15^***^	1.188±0.02^***^
	**Urine**	2.36±0.18	0.3±0.06^###^	0.89±0.05^ NS^	1.11±0.05^*^	1.811±0.02^***^	1.4±0.24^**^	2.0±0.06^***^
**Blood Urea Nitrogen (mg/dl)**	**Serum**	27.59±1.9	49.45±0.7^###^	34.15±1.4^**^	37.7±1.7^**^	33.93±1.3^**^	29.97±1.7^***^	28.30±1.05^***^
**Urea (mg/dl)**	**Serum**	24.04±1.4	54.30±1.0^###^	48.95±1.3^NS^	45.89±1.3^*^	27.04±0.5^***^	32.46±2.1^***^	29.8±0.4^***^
	**Urine**	70.54±2	34.8±1.5^###^	47.2±1.5^*^	65.7±1^***^	55.5±1.8^***^	67.65±1.9^***^	67.61±1^***^
**Uric Acid (mg/dl)**	**Serum**	2.74±0.2	5.45±0.2^###^	4.64±0.08^*^	3.9±0.03^**^	3.39±0.13^***^	2.74±0.08^***^	2.78±0.15^***^
	**Urine**	5.9±0.3	2.25±0.4^###^	2.99±0.13^NS^	4.19±0.16^*^	5.7±0.21^***^	5.64±0.52^**^	5.03±0.09^**^
**Liver function test**								
**Total Protein (gm/dl)**	**Serum**	6.89±0.05	3.45±0.20^###^	4.34±0.21^NS^	5.44±0.21^**^	6.44±0.18^***^	5.89±0.06^***^	6.19±0.26^***^
	**Urine**	2.68±0.15	5.45±0.2^###^	4.24±0.11^*^	3.39±0.16^***^	4.05±0.21^**^	3.23±0.08^***^	3.06±0.2^***^
**Albumin (gm/dl)**	**Serum**	7.89±0.05	6.20±0.03^###^	6.52±0.05^ NS^	6.91±0.05^**^	7.56±0.08^***^	7.65±0.1^***^	7.56±0.1^***^
	**Urine**	6.68±1.1	71.2±2.0^###^	11.02±0.5^***^	8.41±0.4^***^	7.06±0.4^***^	7.15±0.6^***^	7.06±0.3^***^
**Globulin (gm/dl)**	**Serum**	4.46±0.1	7.52±0.3^###^	6.25±0.11^*^	4.44±0.21^***^	5.33±0.29^**^	4.39±0.14^***^	4.64±0.28^***^
**Direct Bilirubin (mg/dl)**	**Serum**	0.03±0.009	0.1±0.009^##^	0.08±0.01^NS^	0.04±0.003^*^	0.06±0.006^NS^	0.02±0.05^**^	0.04±0.009^*^
**Total Bilirubin (mg/dl)**	**Serum**	0.13±0.02	0.29±0.06^##^	0.24±0.09^NS^	0.15±0.6^**^	0.19±0.1^*^	0.16±0.04^**^	0.15±0.02^**^
**ALP (U/L)**	**Serum**	137.4±1.5	194.4±2.1^###^	173.8±2.8^**^	169.2±1.4^***^	149.5±4.2^***^	159.8±2.8^***^	148.0±1.8^***^
**ASAT (U/L)**	**Serum**	168.7±2.5	211.7±2.9^###^	205.4±2.7^ NS^	193.2±2.1^**^	172.8±3.1^***^	170±3.2^***^	167.3±1.2^***^
**ALAT (U/L)**	**Serum**	29.7±1.8	67.08±1.7^###^	57.78±2^*^	54.5±1.8^**^	32.52±1.9^***^	42.15±1.5^***^	32.96±0.7^***^
**Electrolyte**								
**Calcium (mg/dl)**	**Serum**	40.05±2.1	22.7±0.83^###^	29.49±1.14^NS^	34.71±0.87^**^	37.20±1.6^**^	39.07±0.9^***^	37.08±1.1^**^
	**Urine**	4.0±0.1	7.7±0.16^###^	6.2±0.1^*^	4.7±0.12^***^	5.69±0.15^**^	4.56±0.4^***^	4.07±0.1^***^
**Phosphorus (mg/dl)**	**Serum**	6.7±0.2	2.1±0.6^###^	3.5±0.27^ NS^	6.09±0.56^**^	5.44±0.18^**^	5.12±0.5^*^	6.45±0.18^**^
	**Urine**	2.14±0.19	5.28±0.16^###^	3.85±0.1^**^	2.64±0.09^***^	3.2±0.02^***^	2.68±0.15^***^	2.25±0.07^***^
**Magnesium (mg/dl)**	**Serum**	38.63±0.9	23.59±1.0^###^	29.14±1.5^NS^	33.57±1.7^**^	38.16±0.2^***^	33.80±1.1^**^	39.22±0.5^***^
	**Urine**	26.54±0.88	52.89±1.6^###^	47.95±2.7^NS^	35.56±2.9^**^	29.9±1.5^**^	27.13±2^***^	27.18±2.5^***^
**Sodium (mmol/l)**	**Serum**	153.6±0.8	117.1±1.5^###^	114±0.48^NS^	127.1±1.4^**^	150.2±0.5^***^	154.3±1^***^	148.2±0.46^***^
	**Urine**	93.05±2.5	133.0±2.6^###^	122.5±1.7^ NS^	113.3±2^**^	96.27±2^***^	111.4±1^**^	100.5±1.2^***^
**Potassium (mmol/l)**	**Serum**	5.62±0.25	2.58±0.39^###^	4.05±0.1^*^	5.57±0.2^***^	4.47±0.3^*^	5.05±0.18^**^	5.49±0.23^***^
	**Urine**	3.17±0.11	1.19±0.02^###^	2.07±0.1^*^	3.01±0.62^***^	2.61±0.25^**^	3.0±0.13^***^	2.9±0.04^***^

Data are expressed as means ± Standard error of mean (n=8 rats/group). One-way ANOVA was used for statistical analysis. [G-I (Control), G-II (Negative control), G-III (low dose), G-IV (medium dose), G-V (high dose, G-VI (Positive control I), and G-VII (Positive control II)]

G-II was compared with G-I, ###*P*<0.001, ##*P*<0.01, #*P*<0.05 and all the other groups were compared with G-II. ***P<0.001, **P<0.01, and *P<0.05 were considered significant and NS: non-significant

CP: Cisplatin

Data are expressed as means ± standard error of mean (n=8 rats/group). One-way ANOVA was used for statistical analysis [G-I (Normal Control), G-II (Toxic control), G-III (Treatment-Low dose), G-IV (Treatment-Medium dose), G-V (Treatment-High dose), G-VI (Positive control-I), and G-VII (Positive control-II)]

CP: Cisplatin

Data are expressed as means ± standard error of mean (n=8 rats/group). One-way ANOVA was used for statistical analysis. [G-I (Normal Control), G-II (Toxic control), G-III (Treatment-Low dose), G-IV (Treatment-Medium dose), G-V (Treatment-High dose), G-VI (Positive control-I), and G-VII (Positive control-II)]

G-II was compared with G-I, ###*P*<0.001, ##*P*<0.01, #*P*<0.05, and all the other groups were compared with G-II. ***P<0.001, **P<0.01, and *P<0.05 were considered significant, and NS: Non-significant

CP: Cisplatin

CP: Cisplatin

## Discussion

Natural products obtained from medicinal plants have remained popular in developing countries as the primary source of healthcare. This has been attributed primarily to the popularity of traditional medicines. In traditional systems specifically Ayurveda and Unani, *G. glabra *exhibits various pharmacological activities including, respiratory ailments, kidney, metabolic disorders, etc. (Anonymous, 2001 and Anonymous, 2007). However, to provide wider acceptability to traditional herbal medicines there is an unmet need to generate robust scientific data in support of traditional claims. Also, various recent scientific studies are available for its pharmacological effects including anti-oxidant, anti-inflammatory (36), anti-microbial (37), anti-melanogenic, anti-hyperlipidemic, anti-hyperglycemic (38), and neuroprotective (39).

For centuries, herbs have been used traditionally in the treatment of various diseases, playing a vital role in modern medicines. Herbs are thought to act by multifunctional mechanisms due to presence of multiple bioactive constituents that have various pharmacological properties. Oxidative, inflammatory, and apoptotic stress are the major mechanism responsible for CP-induced nephrotoxicity which causes deterioration of kidney functions (40). Thus, it is presupposed that the use of medicinal plants with reported anti-oxidant properties and reversing of inflammatory as well as apoptotic stress leads to a decrease in CP-induced nephrotoxicity (31). 

The present study revealed the nephroprotective effect of GGE against CP-induced nephrotoxicity *in vitro *and *in vivo*. Although *G. glabra* has been consumed by humans, no scientific studies have systematically examined its nephroprotective potential against CP-induced toxicity. In this study, GGE was analyzed through HPTLC and UPLC-MS followed by a demonstration of its nephroprotective activity and the mechanism involved. Specifically, among different parts, roots have been reported to exert the best potential in reverse inflammatory stress (14). Also, the presence of different bioactive constituents reported by HPLTC and UPLC-MS lends a plethora of pharmacological actions such as anti-oxidant and anti-apoptotic activity (7). Herbal medicine with a quality control profile is important for improving its regulatory acceptability (41). Quantitative estimation of markers using HPTLC can be used for quality control analysis of extract. The nephroprotective potential of GGE may be due to the presence of glycyrrhizin, glabridin, liquiritin, flavonoids, and phenolic metabolites (42). Glycyrrhizin (Rt:6.017 ) has been previously found to have anti-cancer, anti-parasitic, anti-inflammatory, anti-oxidative, antibacterial, nephroprotective, and hepatoprotective potential (43-45). Glabridin (Rt:8.832) has been reported to attenuate oxidative stress as well as have anti-inflammatory, anti-tumorigenic, anti-nephritic, antibacterial, and anti-atherogenic effects (46). Another study provides scientific proof for the anti-inflammatory activity of glycyrrhizic acid and liquiritin (Rt:3.152) by modulating the elevated levels of iNOS, COX-2, TNF-α, IL-1β, and IL-6 *in vitro* and *in vivo* (47). Also, liquiritin down-regulated the Bcl-2/Bax ratio and inhibits VEGF expression as well as phosphorylation of JNK and P38 thus, reducing inflammation and restraining angiogenesis (48). Liquiritigenin (Rt: 3.034), is reported to ameliorate renal inflammation through suppression of AQP4/NF-kB/IkBa signaling as well as NLRP3 inflammasome in hyperuricemic rats, thus showing anthyperuricemic effects (49). According to Guan, liquiritin apioside may act as an anti-oxidant and anti-inflammatory, as well as exhibit neuroprotective potential by modulating TGF-β, TNF-α, GSH, and apoptosis (50). Recently studies illustrated the anti-oxidant as well as neuroprotective mechanism of isoliquiritigenin. The compound modulated the SOD, GPx, CAT activities as well as lactate dehydrogenase release and ROS generation. Also, isoliquiritigenin (Rt:4.298) inhibits apoptosis by ameliorating mitochondrial membrane potential loss and nucleus morphology changes (51). Thus, the nephroprotective potential of GGE may be attributed to the presence of various potent bioactive compounds specifically through anti-oxidant, anti-inflammatory, and apoptosis mechanisms.

CP is prescribed for the treatment of multiple cancers, as the first-line drug is platinum-based. CP is widely used as an anti-cancer drug however its use has been limited by severe dose-dependent toxicity (52). Previous studies reported that the use of CP causes marked kidney dysfunction as verified by the altered level of kidney and liver biomarkers (28). Various studies reported nephrotoxicity on exposure to CP caused by oxidative stress, inflammation, as well as apoptosis. Thus, strategies focusing on diverse mechanisms including anti-oxidant, anti-inflammatory, and anti-apoptosis activity to improve CP-induced nephrotoxicity were preferred. Based on traditional uses, phytochemical compositions and anti-oxidant, as well as anti-inflammatory activity of *G. glabra *are reported (53)*. *The present study was conducted for evaluation of the nephroprotective effects of GGE against CP-induced nephrotoxicity in HEK-293 cells and Wistar rats. 

Notably, the study revealed that GGE has no cytotoxic effect in HEK-293 cells. However, cells incubated with CP showed increased cell death along with changes in cellular morphology. Co-treatment of cells with CP and GGE resulted in a significant increase in cell growth as compared with the toxic group highlighting the cytoprotective activity of GGE. Also, GGE showed a significant nephroprotective effect in CP-induced nephrotoxicity in the rodent model. Three doses 31.5, 63, and 126 mg/kg were used for the treatment of CP-induced nephrotoxicity as calculated from Ayurvedic pharmacopoeia (Anonymous, 2001). Ascorbic acid and α-ketoanalogue were used as a positive control as reported by previous scientific studies for its nephroprotective potential through modulation of oxidative, inflammatory, and apoptotic stress (54, 55). 

Our findings demonstrated that GGE modulates the altered levels of anti-oxidant, anti-inflammatory, anti-apoptosis, kidney, and liver parameters as compared with the CP-induced toxicity group. CP also elevates electrolyte levels which cause renal failure (28).

The kidney damage markers including creatinine, BUN, uric acid, and urea are elevated by CP and proven to be restored by administration of GGE. Also, results showed that GGE modulates calcium, potassium, sodium, and magnesium levels. CP leads to kidney harm as well as alteration in functions of other organs specifically, the liver as evidenced by elevated levels of hepatic biomarkers ASAT, ALAT, ALP, and bilirubin levels (56). The current research showed that GGE with CP significantly attenuated the nephrotoxicity as well as hepatotoxicity as compared with the TC group.

Oxidative stress caused due to CP leads to generation of more reactive oxygen which causes diminished anti-oxidant defense activity (57, 58). In the present study, high MDA levels highlight the extension of lipid peroxidation in the CP-induced group, however, GGE with CP significantly reversed altered MDA levels. Presumably, CP diminished the anti-oxidant activity in the cortex and medulla of the kidney, as observed by lowering anti-oxidant enzymes mainly CAT, SOD, and GSH-Px and our result showed GGE significantly increased the levels of CAT and GSH as compared with CP-induced nephrotoxicity group. Also, TNF-α and IL-1β, well-known critical markers of inflammatory disorders have been shown to be altered in CP-induced nephrotoxicity. Further, caspase-3 is considered a reliable as well as sensitive apoptosis marker which is an important contributor to the pathogenesis of CP-induced nephrotoxicity (59). A previous study reported that *G. glabra *significantly reduced the raised levels of inflammatory markers (IL-1,6,12,18; IFN-γ; and TNF-α), ALP, ALT, AST, GGT, urea, creatinine, and metabolic markers preventing gastro duodenal ulcers as well as indicated nephro-, hepato-, hemato-, and immune-protective properties (60). In our study, it appears that oxidative, inflammatory, and apoptosis markers alteration was modulated by GGE significantly. Nassan *et al*. reported that *G. glabra* reversed the gentamicin-induced kidney damages due to anti-oxidant, anti-inflammatory, anti-apoptotic, and cytoprotective properties. *G. glabra* modulated the levels of Gpx, SOD, cytokines (IL-1β and IL-6), renal biomarkers, as well as expression of HO-1, Nrf-2, Cox-2, and Bax, (14). Another study reported that *G. glabra* ameliorates carbon tetrachloride-induced nephrotoxicity by modulating renal biomarkers, anti-oxidant enzymes, as well as histopathological parameters (11).

Our histopathological findings revealed morphological changes in proximal as well as distal convoluted tubules which indicates tubular necrosis compared with previously conducted study (32). The sample of kidney tissue of the treatment group exhibited minimal tubular necrosis and normal glomeruli structure as compared with the TC group. However, medium and high-dose showed significant protection when orally administered before and after CP-induced toxicity. 

The overall study showed that GGE exhibits strong nephroprotective potential. In response to CP-induced nephrotoxicity, different oxidative, inflammatory, and apoptosis (caspase-3) markers were altered. Oral administration of GGE produced a significant protective effect against CP-induced nephrotoxicity by attenuating the elevated levels of kidney and liver markers as well as oxidative damage, anti-inflammatory cytokines, and histopathological changes. The presence of various phytoconstituents with anti-oxidant, anti-inflammatory, and apoptotic roles in GGE may have nephroprotective potential against CP-induced renal damage. 

## Conclusion

The quality control analysis of GGE using HPTLC and UPLC-MS resulted in the generation of scientific data which may be used for detecting its quality, as well as for predicting its mechanism based on the phytoconstituents identified. The results of GGE against CP-induced nephrotoxicity *in vitro *and *in vivo* showed a significant reversal of oxidative stress morphological, biochemical, and histological alterations caused by CP. The effect of GGE was found to be statistically equivalent to standard ascorbic acid and α-ketoanalogue. The future therapeutic use of GGE as nephroprotective warrants further *in silico, in vivo,* as well as clinical investigations to determine the molecular mechanism of its bioactive constituents. Also, the research findings clearly indicate an opportunity for development of potent nephroprotective phytopharmaceuticals in the future from this medicinal plant for the management of kidney and its related complications.

## Authors’ Contributions

SA Contributed to conceptualization, investigation, data interpretation, and article critical evaluation. PB Contributed to conceptualization, literature review, experimental studies, data curation, data interpretation, and writing the original draft. SZ Contributed to data interpretation and article preparation. MUK and GG Contributed to experimental studies and data curation. BJ Contributed to statistical analysis. RP and MAK Contributed to investigation and article preparation.

## Ethical Approval

Animals were obtained from the Central Animal House Facility of Jamia Hamdard which was approved (Approval Number: 1829) by Institutional Animal Ethics Committee (IAEC), Jamia Hamdard, New Delhi, India (Registration No.: 173/GO/RE/S/2000/CPCSEA).

## Conflicts of Interest

The authors declare no conflicts of interest.
